# Supplementary material for: Positive and Negative Design in Stability and Thermal Adaptation of Natural Proteins
Source: PLoS Comput Biol. 2007 Mar 23;3(3):e52. doi: 10.1371/journal.pcbi.0030052 (PMC1829478; doi:10.1371/journal.pcbi.0030052)
Supplement: Supplementary file 1 [file pcbi.0030052.sg001.doc]

**Figure S1.** Average melting temperature of designed lattice proteomes (5000 sequences each) depending on the environmental temperature $T_{env}$entering the P-design procedure (2000 Monte Carlo mutation steps to generate each sequence).
