# Supplementary material for: Positive and Negative Design in Stability and Thermal Adaptation of Natural Proteins
Source: PLoS Comput Biol. 2007 Mar 23;3(3):e52. doi: 10.1371/journal.pcbi.0030052 (PMC1829478; doi:10.1371/journal.pcbi.0030052)
Supplement: Supplementary file 2 [file pcbi.0030052.sg002.doc]

**A**

**B**

**Figure S2.** Temperature dependences of amino acid fraction for Val (**A**) and Glu (**B**) in 204 natural psycho-, meso, thermo, and hyperthermophilic proteomes (habitat temperatures from -10 to +110 ºC, see Table S1 for optimal growth temperatures and references).
