# Supplementary material for: Positive and Negative Design in Stability and Thermal Adaptation of Natural Proteins
Source: PLoS Comput Biol. 2007 Mar 23;3(3):e52. doi: 10.1371/journal.pcbi.0030052 (PMC1829478; doi:10.1371/journal.pcbi.0030052)
Supplement: Supplementary file 3 [file pcbi.0030052.st001.doc]

**Supporting Information: Table S1 and References**

**Legend to Table S1.** Prokaryotes with completely sequenced genomes and their optimal growth temperatures. The columns are: **NN**, number; **Organism**, name of the organism; **OGT**, optimal growth temperature, °C; **Source**, source of the optimal growth temperature.

**ATCC**: American Type Culture Collection, accessed at [http://www.atcc.org](http://www.atcc.org/)

**DSMZ**: German Collection of Microorganisms and Cell Cultures,

accessed at [http://www.dsmz.de](http://www.dsmz.de/)

**PGTdb**: The Prokaryotic Growth Temperature Database,

accessed at [http://pgtdb.csie.ncu.edu.tw](http://pgtdb.csie.ncu.edu.tw/)

Reference: S. L. Huang, L. C. Wu, H. K. Laing, K.T. Pan, and J. T. Horng, *Bioinformatics*, Vol. 20, pp. 276-278, 2004.

The complete genomes were downloaded from the NCBI Genome database at <http://www.ncbi.nih.gov/entrez/query.fcgi?db=Genome>

**Table S1**

| **NN** | **Organism** | **OGT, °C** | **Source** |
| --- | --- | --- | --- |
| 1 | *Acidobacteria bacterium Ellin345* | 25 | [1] |
| 2 | *Aeropyrum pernix* | 95 | [2] |
| 3 | *Anabaena variabilis ATCC 29413* | 35 | [3] |
| 4 | *Anaeromyxobacter dehalogenans 2CP-C* | 27 | ATCC |
| 5 | *Aquifex aeolicus* | 85 | [4] |
| 6 | *Archaeoglobus fulgidus* | 83 | [2] |
| 7 | *Bordetella bronchiseptica* | 36 | DSMZ, [5] |
| 8 | *Borrelia burgdorferi* | 29 | [5] |
| 9 | *Campylobacter jejuni* | 40 | DSMZ, PGTdb |
| 10 | *Carboxydothermus hydrogenoformans Z-2901* | 67 | DSMZ |
| 11 | *Chlorobium tepidum TLS* | 48 | [6] |
| 12 | *Colwellia psychrerythraea 34H* | 8 | [7] |
| 13 | *Corynebacterium glutamicum ATCC 13032 Bielefeld* | 33 | DSMZ, ATCC |
| 14 | *Deinococcus geothermalis DSM 11300* | 47 | DSMZ |
| 15 | *Desulfitobacterium hafniense Y51* | 38 | [8] |
| 16 | *Desulfotalea psychrophila LSv54* | 10 | [9,10] |
| 17 | *Erythrobacter litoralis HTCC2594* | 29 | DSMZ, ATCC |
| 18 | *Francisella tularensis tularensis* | 35 | [11] |
| 19 | *Geobacillus kaustophilus HTA426* | 60 | [12] |
| 20 | *Geobacter metallireducens GS-15* | 32 | [13] |
| 21 | *Geobacter sulfurreducens* | 32 | [14] |
| 22 | *Haemophilus ducreyi 35000HP* | 32 | DSMZ, ATCC |
| 23 | *Lactobacillus acidophilus NCFM* | 41 | DSMZ, ATCC |
| 24 | *Leifsonia xyli xyli CTCB0* | 29 | DSMZ, ATCC |
| 25 | *Methanobacterium thermoautotrophicum* | 65 | [15] |
| 26 | *Methanococcoides burtonii DSM 6242* | 23 | DSMZ |
| 27 | *Methanococcus jannaschii* | 85 | [2] |
| 28 | *Methanococcus maripaludis S2* | 35 | [16] |
| 29 | *Methanopyrus kandleri* | 98 | [2] |
| 30 | *Methanosarcina acetivorans* | 40 | [17] |
| 31 | *Methanosarcina barkeri fusaro* | 35 | DSMZ, ATCC |
| 32 | *Methanosarcina mazei* | 36 | DSMZ, ATCC |
| 33 | *Methanospirillum hungatei JF-1* | 35 | DSMZ, PGTdb |
| 34 | *Moorella thermoacetica ATCC 39073* | 57 | DSMZ, ATCC |
| 35 | *Mycobacterium avium paratuberculosis* | 39 | DSMZ, PGTdb |
| 36 | *Nanoarchaeum equitans* | 82 | [18] |
| 37 | *Natronomonas pharaonis* | 41 | DSMZ, PGTdb |
| 38 | *Nitrobacter hamburgensis X14* | 28 | DSMZ |
| 39 | *Nitrobacter winogradskyi Nb-255* | 27 | DSMZ, ATCC |
| 40 | *Oceanobacillus iheyensis* | 28 | DSMZ |
| 41 | *Pelodictyon luteolum DSM 273* | 25 | DSMZ |
| 43 | *Photobacterium profundum SS9* | 15 | [19] |
| 44 | *Photorhabdus luminescens* | 29 | DSMZ, ATCC |
| 45 | *Picrophilus torridus DSM 9790* | 60 | [20] |
| 46 | *Pseudomonas aeruginosa* | 40 | [5] |
| 47 | *Pseudomonas fluorescens Pf-5* | 32 | [5] |
| 48 | *Pseudomonas putida KT2440* | 28 | [10] |
| 49 | *Psychrobacter arcticum 273-4* | 22 | [21] |
| 50 | *Pyrobaculum aerophilum* | 100 | [22] |
| 51 | *Pyrococcus abyssi* | 96 | [23] |
| 52 | *Pyrococcus furiosus* | 100 | [2] |
| 53 | *Pyrococcus horikoshii* | 98 | [24] |
| 54 | *Rhizobium etli CFN 42* | 27 | DSMZ, ATCC |
| 55 | *Rhodoferax ferrireducens DSM 15236* | 25 | DSMZ |
| 56 | *Rhodospirillum rubrum ATCC 11170* | 27 | DSMZ, PGTdb |
| 57 | *Rickettsia conorii* | 35 | PGTdb |
| 58 | *Rickettsia prowazekii* | 35 | PGTdb |
| 59 | *Rickettsia typhi wilmington* | 35 | PGTdb |
| 60 | *Saccharophagus degradans 2-40* | 28 | DSMZ |
| 61 | *Shewanella denitrificans OS217* | 20 | DSMZ, ATCC |
| 62 | *Sodalis glossinidius morsitans* | 28 | DSMZ |
| 63 | *Staphylococcus aureus aureus MRSA252* | 34 | [5] |
| 64 | *Streptococcus thermophilus CNRZ1066* | 42 | [5] |
| 65 | *Streptomyces avermitilis* | 27 | DSMZ, ATCC |
| 66 | *Streptomyces coelicolor* | 27 | DSMZ, ATCC |
| 67 | *Sulfolobus acidocaldarius DSM 639* | 80 | [2] |
| 68 | *Sulfolobus solfataricus* | 80 | [25,26] |
| 69 | *Sulfolobus tokodaii* | 80 | [27] |
| 70 | *Symbiobacterium thermophilum IAM14863* | 60 | [28] |
| 71 | *Synechococcus elongatus PCC 7942* | 26 | ATCC |
| 72 | *Thermoanaerobacter tengcongensis* | 75 | [29] |
| 73 | *Thermobifida fusca YX* | 57 | [30] |
| 74 | *Thermococcus kodakaraensis KOD1* | 95 | [31,32] |
| 75 | *Thermoplasma acidophilum* | 59 | [25,33] |
| 76 | *Thermoplasma volcanium* | 60 | [25,34] |
| 77 | *Thermosynechococcus elongatus* | 55 | [35] |
| 78 | *Thermotoga maritima* | 80 | [2] |
| 79 | *Thermus thermophilus HB27* | 85 | [36] |
| 80 | *Thiomicrospira crunogena XCL-2* | 25 | DSMZ, ATCC |
| 81 | *Vibrio fischeri ES114* | 28 | ATCC, PGTdb |
| 82 | *Yersinia pestis KIM* | 28 | [37] |
| 83 | *Zymomonas mobilis ZM4* | 27 | DSMZ, ATCC |

**References:**

1. Janssen PH, Yates PS, Grinton BE, Taylor PM, Sait M (2002) Improved culturability of soil bacteria and isolation in pure culture of novel members of the divisions Acidobacteria, Actinobacteria, Proteobacteria, and Verrucomicrobia. Appl Environ Microbiol 68: 2391-2396.

2. Stetter KO (1999) Extremophiles and their adaptation to hot environments. FEBS Lett 452: 22-25.

3. Kratz WA, J. Myers (1955) Nutrition and growth of several blue-green algae. American Journal of Botany 42: 282-287.

4. Deckert G, Warren PV, Gaasterland T, Young WG, Lenox AL, et al. (1998) The complete genome of the hyperthermophilic bacterium Aquifex aeolicus. Nature 392: 353-358.

5. (1974) Bergey's Manual of Determinative Bacteriology. Baltimore: The Williams & Wilkins Company.

6. Eisen JA, Nelson KE, Paulsen IT, Heidelberg JF, Wu M, et al. (2002) The complete genome sequence of Chlorobium tepidum TLS, a photosynthetic, anaerobic, green-sulfur bacterium. Proc Natl Acad Sci U S A 99: 9509-9514.

7. Methe BA, Nelson KE, Deming JW, Momen B, Melamud E, et al. (2005) The psychrophilic lifestyle as revealed by the genome sequence of Colwellia psychrerythraea 34H through genomic and proteomic analyses. Proc Natl Acad Sci U S A 102: 10913-10918.

8. Bouchard B, Beaudet R, Villemur R, McSween G, Lepine F, et al. (1996) Isolation and characterization of Desulfitobacterium frappieri sp. nov., an anaerobic bacterium which reductively dechlorinates pentachlorophenol to 3-chlorophenol. Int J Syst Bacteriol 46: 1010-1015.

9. Knoblauch C, Sahm K, Jorgensen BB (1999) Psychrophilic sulfate-reducing bacteria isolated from permanently cold arctic marine sediments: description of Desulfofrigus oceanense gen. nov., sp. nov., Desulfofrigus fragile sp. nov., Desulfofaba gelida gen. nov., sp. nov., Desulfotalea psychrophila gen. nov., sp. nov. and Desulfotalea arctica sp. nov. Int J Syst Bacteriol 49 Pt 4: 1631-1643.

10. Rabus R, Ruepp A, Frickey T, Rattei T, Fartmann B, et al. (2004) The genome of Desulfotalea psychrophila, a sulfate-reducing bacterium from permanently cold Arctic sediments. Environ Microbiol 6: 887-902.

11. Payne MP, Morton RJ (1992) Effect of culture media and incubation temperature on growth of selected strains of Francisella tularensis. J Vet Diagn Invest 4: 264-269.

12. Takami H, Takaki Y, Chee GJ, Nishi S, Shimamura S, et al. (2004) Thermoadaptation trait revealed by the genome sequence of thermophilic Geobacillus kaustophilus. Nucleic Acids Res 32: 6292-6303.

13. Lovley DR, Giovannoni SJ, White DC, Champine JE, Phillips EJ, et al. (1993) Geobacter metallireducens gen. nov. sp. nov., a microorganism capable of coupling the complete oxidation of organic compounds to the reduction of iron and other metals. Arch Microbiol 159: 336-344.

14. Caccavo F, Jr., Lonergan DJ, Lovley DR, Davis M, Stolz JF, et al. (1994) Geobacter sulfurreducens sp. nov., a hydrogen- and acetate-oxidizing dissimilatory metal-reducing microorganism. Appl Environ Microbiol 60: 3752-3759.

15. Smith DR, Doucette-Stamm LA, Deloughery C, Lee H, Dubois J, et al. (1997) Complete genome sequence of Methanobacterium thermoautotrophicum deltaH: functional analysis and comparative genomics. J Bacteriol 179: 7135-7155.

16. Haney PJ, Badger JH, Buldak GL, Reich CI, Woese CR, et al. (1999) Thermal adaptation analyzed by comparison of protein sequences from mesophilic and extremely thermophilic Methanococcus species. Proc Natl Acad Sci U S A 96: 3578-3583.

17. Sowers KR, Baron, S.F., Ferry J.G. (1984) *Methanosracina acetivorans* sp. nov., and Acetotrophic Methane-Producing Becterium Isolated from Marine Sediments. Applied and Environmental Micribiology 47: 971-978.

18. Huber H, Hohn MJ, Stetter KO, Rachel R (2003) The phylum Nanoarchaeota: present knowledge and future perspectives of a unique form of life. Res Microbiol 154: 165-171.

19. Bartlett DH (2002) Pressure effects on in vivo microbial processes. Biochim Biophys Acta 1595: 367-381.

20. Futterer O, Angelov A, Liesegang H, Gottschalk G, Schleper C, et al. (2004) Genome sequence of Picrophilus torridus and its implications for life around pH 0. Proc Natl Acad Sci U S A 101: 9091-9096.

21. Bakermans C, Ayala-Del-Rio HL, Ponder MA, Vishnivetskaya T, Gilichinsky D, et al. (2006) Psychrobacter cryohalolentis sp. nov. and Psychrobacter arcticus sp. nov., isolated from Siberian permafrost. Int J Syst Evol Microbiol 56: 1285-1291.

22. Fitz-Gibbon ST, Ladner H, Kim UJ, Stetter KO, Simon MI, et al. (2002) Genome sequence of the hyperthermophilic crenarchaeon Pyrobaculum aerophilum. Proc Natl Acad Sci U S A 99: 984-989.

23. Cohen GN, Barbe V, Flament D, Galperin M, Heilig R, et al. (2003) An integrated analysis of the genome of the hyperthermophilic archaeon Pyrococcus abyssi. Mol Microbiol 47: 1495-1512.

24. Kawarabayasi Y, Sawada M, Horikawa H, Haikawa Y, Hino Y, et al. (1998) Complete sequence and gene organization of the genome of a hyper-thermophilic archaebacterium, Pyrococcus horikoshii OT3. DNA Res 5: 55-76.

25. Schafer G, Engelhard M, Muller V (1999) Bioenergetics of the Archaea. Microbiol Mol Biol Rev 63: 570-620.

26. She Q, Singh RK, Confalonieri F, Zivanovic Y, Allard G, et al. (2001) The complete genome of the crenarchaeon Sulfolobus solfataricus P2. Proc Natl Acad Sci U S A 98: 7835-7840.

27. Kawarabayasi Y, Hino Y, Horikawa H, Jin-no K, Takahashi M, et al. (2001) Complete genome sequence of an aerobic thermoacidophilic crenarchaeon, Sulfolobus tokodaii strain7. DNA Res 8: 123-140.

28. Ohno M, Shiratori H, Park MJ, Saitoh Y, Kumon Y, et al. (2000) Symbiobacterium thermophilum gen. nov., sp. nov., a symbiotic thermophile that depends on co-culture with a Bacillus strain for growth. Int J Syst Evol Microbiol 50 Pt 5: 1829-1832.

29. Xue Y, Xu Y, Liu Y, Ma Y, Zhou P (2001) Thermoanaerobacter tengcongensis sp. nov., a novel anaerobic, saccharolytic, thermophilic bacterium isolated from a hot spring in Tengcong, China. Int J Syst Evol Microbiol 51: 1335-1341.

30. Bonamore A, Ilari A, Giangiacomo L, Bellelli A, Morea V, et al. (2005) A novel thermostable hemoglobin from the actinobacterium Thermobifida fusca. Febs J 272: 4189-4201.

31. Atomi H, Fukui T, Kanai T, Morikawa M, Imanaka T (2004) Description of Thermococcus kodakaraensis sp. nov., a well studied hyperthermophilic archaeon previously reported as Pyrococcus sp. KOD1. Archaea 1: 263-267.

32. Fujiwara S, Takagi M, Imanaka T (1998) Archaeon Pyrococcus kodakaraensis KOD1: application and evolution. Biotechnol Annu Rev 4: 259-284.

33. Ruepp A, Graml W, Santos-Martinez ML, Koretke KK, Volker C, et al. (2000) The genome sequence of the thermoacidophilic scavenger Thermoplasma acidophilum. Nature 407: 508-513.

34. Kawashima T, Amano N, Koike H, Makino S, Higuchi S, et al. (2000) Archaeal adaptation to higher temperatures revealed by genomic sequence of Thermoplasma volcanium. Proc Natl Acad Sci U S A 97: 14257-14262.

35. Nakamura Y, Kaneko T, Sato S, Ikeuchi M, Katoh H, et al. (2002) Complete genome structure of the thermophilic cyanobacterium Thermosynechococcus elongatus BP-1. DNA Res 9: 123-130.

36. Henne A, Bruggemann H, Raasch C, Wiezer A, Hartsch T, et al. (2004) The genome sequence of the extreme thermophile Thermus thermophilus. Nat Biotechnol 22: 547-553.

37. Bibel DJ, Chen TH (1976) Diagnosis of plaque: an analysis of the Yersin-Kitasato controversy. Bacteriol Rev 40: 633-651.
